# Diagnosis of systemic toxoplasmosis with HIV infection using DNA extracted from paraffin-embedded tissue for polymerase chain reaction: a case report

**DOI:** 10.1186/1752-1947-4-265

**Published:** 2010-08-11

**Authors:** Yoichiro Okubo, Minoru Shinozaki, Sadako Yoshizawa, Haruo Nakayama, Megumi Wakayama, Tsutomu Hatori, Aki Mituda, Takayuki Hirano, Kayoko Shimodaira, Zhi Yuzhu, Kazutoshi Shibuya

**Affiliations:** 1 Department of Surgical Pathology, Toho University School of Medicine, 6-11-1 Omori-Nishi, Ota-Ku, Tokyo, 143-8541, Japan; 2 Department of Infection Control, Toho University Medical Center, Omori Hospital, 6-11-1 Omori-Nishi, Ota-Ku, Tokyo, 143-8541, Japan

## Abstract

**Introduction:**

Toxoplasmosis can be a life-threatening disease when it occurs in patients with HIV infection. In particular, meningioencephalitis has been regarded as the most common toxoplasmic complication in such patients. However, toxoplasmic meningitis in a patient with HIV infection is extremely rare and purulent or tuberculous meningitis should be considered initially as a disease for differential diagnosis in Japan.

**Case presentation:**

Toxoplasmic meningitis in a patient with HIV infection is reported. A 36-year-old Japanese man presented with fever, pulsating headache, lumbago, nausea, and vomiting. No examinations suggested toxoplasmosis including cerebrospinal fluid examinations, images, and serological tests. The result of a polymerase chain reaction assay using paraffin-embedded section was regarded as the conclusive evidence for the diagnosis.

**Conclusions:**

We wish to emphasize the usefulness of polymerase chain reaction assays with nucleic acid extracted from paraffin-embedded tissue sections processed for routine histopathological examination, if the section shows the infectious agents or findings suggesting some infectious diseases.

## Introduction

*Toxoplasma gondii *is known as one of the most common infectious protozoan parasites that has a worldwide distribution [1-3]. Cats are recognized as the only definitive hosts of *T. gondii*, but humans can be infected by the ingestion of oocysts or tissue cysts [4]. *T. gondii *infection is generally asymptomatic or associated with lymphadenopathy and manifests as a flu-like illness in immunocompetent individuals. However, the infection causes severe and fatal complications, especially in the central nervous system, in immunocompromised individuals [2,5]. This paper describes a case of toxoplasmosis in patient with HIV infection that was diagnosed by polymerase chain reaction (PCR) with the use of nucleic acid extracted from formalin-fixed and paraffin-embedded tissue (bone marrow aspiration clot) sections prepared for routine histopathological examination.

## Case presentation

**Figure 1:**
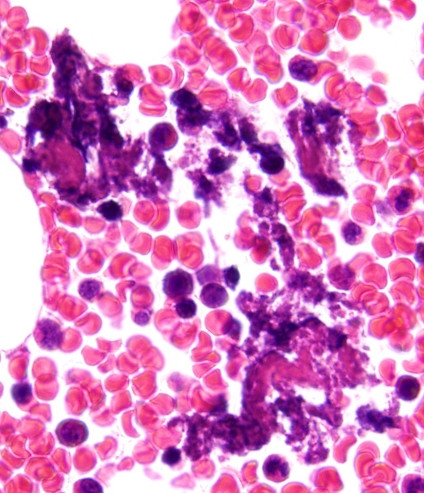
**Hematoxylin and eosin stain of bone marrow**. Hematoxylin and eosin stain of bone marrow. Clustered intra-cellular basophilic granuli suggesting toxoplasmosis was present (x1000).

**Figure 2:**
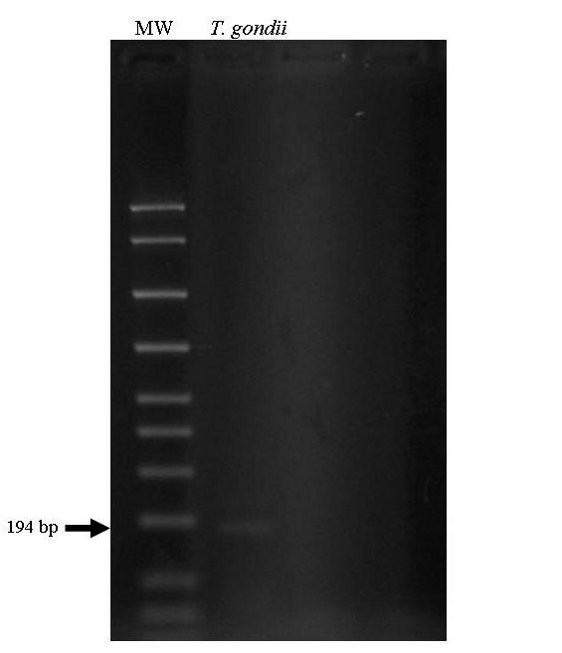
**Result of nested PCR for *T. gondii***. The detection threshold of nested PCR for *T. gondii*. 194 bp was the expected size of nested PCR for *T. gondii *(Abbreviation, MW: molecular weight).

A 36-year-old Japanese man with a 14-month history of HIV infection presented with fever, pulsating headache, lumbago, nausea, and vomiting four week prior to his admission. Although highly active anti-retroviral therapy (HAART) had been started (lamivudine, azidothymidine, and lopinavir plus ritonavir) after completion of treatment for pneumocystis pneumonia, which had been the initial clinical manifestation of our patient, his CD4-positive lymphocyte counts in peripheral blood has never recovered to more than 200 cells/mm^3^. Therefore, three months before admission, abacavir was given instead of azidothymidine, but was also insufficient for increasing CD4-positive lymphocytes. Furthermore, according to the guidelines, prophylaxis against *Pneumocystis jirovecii *had been started. In our case, atovaquone had been administered, because sulfamethoxazole-trimethoprim and pentamidine had caused hepatic and renal insufficiency, respectively. On physical examination, our patient reported headache with neck stiffness. His axillary temperature was 38.2°C. Chest radiography and computed tomography (CT) of the brain showed no abnormalities. He was diagnosed as purulent meningitis, initially, because of an increasing of neutrophils count in cerebrospinal fluid (CSF). Broad-spectrum antimicrobials, however, had no effect on this meningitis. CD4-positive lymphocyte counts 146/μl in peripheral blood. He did not show increasing of immunoglobulin G (IgG) and immunoglobulin M (IgM) fraction of anti-T. gondii antibody (enzyme-linked immunosorbant assay, ELISA). Three weeks after admission, due to worsened headache and lumbago, magnetic resonance imaging (MRI) of the brain and lumbar vertebrae was performed and showed enhanced small nodules at right superior pons and bilateral superior cerebellum, peripheral enhancement at the bilateral superior pons, and enhanced lesions which was parallel to left inner ear. These findings strongly suggested meningitis with granuloma formation, such as tuberculosis. Furthermore, MRI of the lumber vertebrae also suggested the presence of the granulomatous lesion. However, the PCR assay targeting mycobacterium tuberculosis was negative. Therefore, bone marrow aspiration biopsy was performed for histopathological examination to elucidate the causative agent of generalized infection. The specimen, bone marrow aspiration clot, was fixed with 10% formalin and embedded in paraffin wax after dehydration which was cut into 3 μm-thick sections, and routinely stained with hematoxylin and eosin double stain. Histopathological examination indicated hypocellular bone marrow in which clustered intra-cellular basophilic granuli were present (Figure 1). These were confirmed as microcalcification by Von Kossa's stain. Therefore, the PCR assay with toxoplasma-specific primer was performed using nucleic acid extracted from the formalin-fixed and paraffin-embedded tissue (bone marrow aspiration) section. In the procedure, paraffin-embedded tissue sections (bone marrow aspiration clot) were deparaffinized by xylene and immersed in absolute ethanol. The air-dried pellets of dehydrated section were then resuspended in extraction buffer (Tris-HCl [50 mM, pH 8.5], NaCl [50 mM], EDTA [10 mM], sodium dodecyl sulfate [0.5%], proteinase K [10 mg/mL]) at 95°C. The samples were completely submerged in the extraction buffer and incubated at 56°C for 12 hours. The supernatant was then purified by phenol-chloroform extraction and ethanol precipitation, and was resuspended in 25 mL of DNase-free buffer (Tris-HCl [10 mM], EDTA [1 mM]), and was stored at 20°C until use for DNA amplification. PCR for B1 gene of *T. gondii *was carried out following the previous description of Tachikawa *et al. *[6]. The primers used in the assay are summarized in Additional file 1. Conditions of nested PCR for *T. gondii *were 0.5 pmol/L primer, 2.5 mM MgCl_2 _, 0.2 mM dNTP, and 0.02 U/L Taq DNA polymerase. First PCR was performed in thermal cycler starting with a pre-incubation at 94°C for three minutes, followed by 40 PCR cycles of one minute denaturation at 94°C, one minute annealing at 58°C, one minute elongation at 72°C. The first PCR product was added to a new reaction mixture. Compositions and PCR cycles were same as the first PCR. Second PCR product was electrophoresed on a 3% agarose gel. As a result, the specific PCR product of *T. gondii *was obtained from the extract from paraffin-embedded tissue sections of the bone marrow biopsy (Figure 2). Anti*-T. gondii *therapy consisting of pyrimethamine, clindamycin, and leucovorin had been started. After sulfadiazine desensitization, clindamycin was replaced with sulfadiazine. Together with this, although our patient was negative on frequent PCR assay for tuberculosis, anti-tubercular treatment had been continued due to suspected tuberculosis from MRI. According to this, his fever was improved, but other clinical symptoms remained. Finally, anti*-T. gondii *therapy consisting pyrimethamine, sulfadiazine, and leucovorin plus prednisolone had an effect on improving his clinical symptoms and he was referred to other hospital in his home city at his request.

## Discussion

Toxoplasmosis can be a life-threatening disease when it occurs in patients with HIV infection with decreased CD4 positive lymphocytes [7]. In particular, encephalitis is the most common toxoplasmic complication in such individuals [8]. It has been reported that the incidence of toxoplasmic meningioencephalitis ranges from 3 to 50% [9]. However, in Japan, the latest study reported that only 1.07% of patients with HIV develop toxoplasmosis [10] and the seroprevalence of IgG anti-toxoplasma antibodies in Japanese patients was less than 10% [11,12]. Therefore, as toxoplasmic meningitis in patients with HIV infection is extremely rare, purulent or tuberculous meningitis should be considered initially as a disease for differential diagnosis. In our case, neither CSF examinations nor MRI of the brain were typical for toxoplasmosis. Failure of our patient's symptoms to respond to anti-bacterial and anti-tubercular treatment led us to perform bone marrow biopsy to search for another cause of generalized opportunistic infection. Histopathological findings from bone marrow biopsy suggested toxoplasmosis because the evidence of clustered micro-calcifying granules, but this finding was not adequate to diagnose the disease definitively. Therefore, to make the diagnosis, we employed a PCR assay using nucleic acid extracted from formalin-fixed and paraffin-embedded tissue section of the bone marrow. A major and classical diagnostic procedure for toxoplasmosis has been constituent with both serological tests and histopathological examinations [1], but these methods have limitations. In particularly, serological tests often fail to detect *T. gondii *infection in patients with HIV infection due to their decreased functioning of immunoglobulin production [13]. Recently, several PCR assays have been developed with different gene targets. Among them, a detection of *T. gondii *DNA has been regarded as one of the useful diagnostic procedures [1]. Furthermore, the potential of PCR assay to detect *T. gondii *in CSF has been previously reported, in which the sensitivity and specificity were described between 44 and 100%, 94 and 100%, respectively [9]. PCR assay using CSF could be successful to detect gene form *T. gondii*. However, to avoid an invasive diagnostic procedure we employed a PCR assay using nucleic acid extracted from the paraffin-embedded sections, which also had revealed strongly suggestive alterations for the toxoplasmosis. Although there was only a report that referred to PCR assay using nucleic acid extracted from paraffin-embedded tissue sections and this method has not been validated until now [14].

## Conclusions

We wish to emphasize the usefulness of PCR assay using nucleic acid extracted from paraffin-embedded tissue sections processed for routine histopathological examination, if the section shows the infectious agents or findings suggesting some infectious diseases.

## Abbreviations

CSF: cerebrospinal fluid; CT: computed tomography; HAART: highly active anti-retroviral therapy; HIV: human immunodeficiency virus; IgG: immunoglobulin G; MRI: magnetic resonance imaging; PCR: polymerase chain reaction.

## Consent

Written informed consent was obtained from the patient for publication of this case report and any accompanying images. A copy of the written consent is available for review by the Editor-in-Chief of this journal.

## Competing interests

KS reports received research grants from Pfizer Japan Inc., Janssen Pharmaceutical K.K., and Dainippon Sumitomo Pharma Co. All authors declare that they have no competing interests.

## Authors' contributions

YO, conceptualized the case report, integrated the data, and wrote the manuscript as a major contributor; MS, carried out the HE stain, Von Kossa's stain, immunohistochemical staining and PCR assay; SY, contributed to management of the patient; HN, MW, TH, AM, and TH, carried out the histopathologic evaluation and revised histopathological description; KS, and ZY, assisted PCR assay; KS, gave final approval to the manuscript as a corresponding author. All authors contributed to conceptualizing and writing this case report.

## Supplementary Material

Additional file 1 **Primers of nested PCR for *****T. gondii***. Click here for file
