# Supplementary figures and images for: Diagnosis of systemic toxoplasmosis with HIV infection using DNA extracted from paraffin-embedded tissue for polymerase chain reaction: a case report
Source: J Med Case Rep. 2010 Aug 11;4:265. doi: 10.1186/1752-1947-4-265 (PMC2924357; doi:10.1186/1752-1947-4-265)

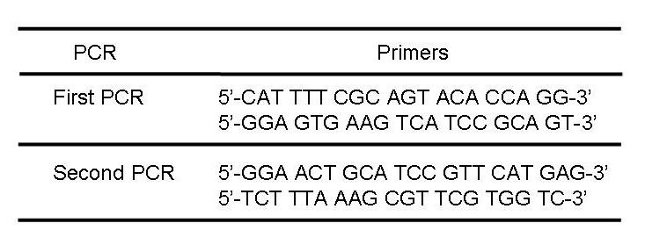

Supplement: Additional file 1 — Primers of nested PCR for T. gondii. [file 1752-1947-4-265-S1.JPEG]
